# Decomposing the effects of changes of population size, age-sex profile, health status and residual factors on growth in hospital activity in English hospitals: an ecological database study from 2011–2019

**DOI:** 10.1186/s12913-025-13662-0

**Published:** 2025-12-17

**Authors:** Steven Wyatt, Gabriel Hobro, Paul Seamer, Mohammed A. Mohammed, Peter Spilsbury

**Affiliations:** 1 The Strategy Unit, Birmingham, UK; 2 New Hospitals Programme, London, NHS UK; 3 University of Bradford, Bradford, UK; 4 Cardiff Business School, University of Cardiff, Cardiff, Wales UK

**Keywords:** Hospital activity decomposition, Population growth, Health status growth, Residual growth

## Abstract

**Background:**

Hospitals are central to healthcare systems and understanding the drivers of hospital activity is critical for effective capacity planning, especially amid demographic shifts and fiscal pressure. In the English National Health Service (NHS), there are plans to construct 40 new hospitals. There is limited evidence on factors driving hospital activity. This study provides retrospective estimates of the effects of population growth (P), changes in the age–sex structure (S), shifts in age-specific health status (H), and residual factors (R) on hospital activity in England.

**Methods:**

Total annual growth is modelled as G = (1 + P)(1 + S)(1 + H)(1 + R) − 1. Negative binomial regression models using hospital episode statistics from 2011 to 2019 were used to decompose total annual growth rates (G) into P,S,H and R by five points of delivery (listed below).

**Results:**

The annual growth rate for elective admissions was 2.29% (95% CI: 1.06% to 3.54%) which was made up of *p* = 0.88% (−0.96% to 2.67%), S = 0.69% (−0.62% to 2.18%), H = −0.03% (−0.23% to 0.17%) and *R* = 0.73% (0.58% to 0.88%). The annual growth rate for non-elective admission was 2.69% (1.55% to 3.83%) which was made up of *p* = 0.59% (−1.08% to 2.50%), S = 0.25% (−1.17% to 1.61%), H = 0.18% (−0.43% to 0.81%) and *R* = 1.65% (1.13% to 2.17%). The annual growth rate for maternity admissions was −0.70% (−5.65% to 4.51%) which was made up of *p* = 0.41% (−6.25% to 8.36%), S = −0.08% (−4.93% to 5.23%), H = 0.00% (NA) and *R* = −1.02% (−1.61% to −0.43%). The annual growth rate for outpatient attendance was 4.51% (3.62% to 5.40%) which was made up of *p* = 0.61% (−0.71% to 1.96%), S = 0.46% (−0.63% to 1.56%), H = −0.02% (−0.43% to 0.38%) and *R* = 3.41% (3.11% to 3.71%). The annual growth rate for emergency department attendance was 1.29% (0.71% to 1.88%) which was made up of *p* = 0.27% (−0.56% to 1.09%), S = 0.09% (−0.55% to 0.70%), H = 0.09% (−0.21% to 0.39%) and *R* = 0.84% (0.58% to 1.10%).

**Conclusions:**

In general, growth in hospital activity was mostly driven by residual (R) factors, followed by population growth (P). Age-specific health status (H) had the lowest impact. These data may provide useful context for planning future hospital activity in England.

**Supplementary Information:**

The online version contains supplementary material available at 10.1186/s12913-025-13662-0.

## Introduction

Hospitals play a central role in healthcare systems, delivering critical diagnostic, therapeutic, and emergency services. As demand for hospital care continues to grow—driven by demographic shifts such as population ageing and constrained by fiscal pressures—understanding the underlying drivers of hospital activity has become essential. This insight is vital for ensuring the sustainability of health systems, particularly given that hospital care represents the most resource-intensive and costly component of healthcare expenditure [1–7].

The Behavioural Model of Health Services Use [8, 9], a widely applied framework for understanding healthcare utilization, classifies the determinants of health service use into three main categories: predisposing characteristics (e.g., age, sex), enabling resources (e.g., availability and accessibility of care), and need factors (both perceived and clinically evaluated health status). This model provides a useful lens for examining how population characteristics and systemic factors shape patterns of hospital use.

In England, the National Health Service (NHS), which has about 515 hospitals [10], is currently undertaking a major capital investment programme, including plans to construct 40 new hospitals [11, 12]. This initiative underscores the importance of planning future hospital capacity to align with the evolving health needs of the population. However, there remains limited empirical evidence on the specific factors driving changes in hospital activity in England. This study aims to address this gap by providing a retrospective population-based, macro-level assessment of the drivers of hospital activity in England, by decomposing annual growth in hospital activity into four components: population growth (P), changes in the age–sex structure (S)—a predisposing factor, shifts in age-specific health status (H)—a proxy for population-level need, and residual factors (R), which includes enabling resources and other factors (not otherwise captured by P,S,H).

We model total growth in hospital activity (G) using a multiplicative decomposition framework, expressed as G = (1 + P)(1 + S)(1 + H)(1 + R) − 1. By quantifying the relative contributions of these components, the study offers retrospective evidence to inform long-term hospital capacity planning in the English NHS.

## Methods

### Population and setting

Our analysis was focused on growth rates of NHS-funded hospital activity in England between 2011 and 2019. The start of the study period was determined by the availability and quality of key datasets. The study period ends in 2019 to avoid the shock associated with the COVID-19 pandemic. We considered five points of delivery of hospital activity - inpatient elective spells, inpatient non-elective spells, inpatient maternity spells, emergency department (ED) attendances at type 1 consultant-led emergency departments, and outpatient attendances. For maternity activity, we limit the sex to females and the age to 15–49 years.

### Data sources

Our analysis dataset was assembled using data from six sources: (1) Hospital Episode Statistics for Admitted Patient Care (HES APC) [13], (2) Hospital Episode Statistics for Outpatient Care (HES OPA), (3) the Emergency Care Dataset (ECDS) [14], (4) mid-year population estimates published by the Office for National Statistics [15], (5) life tables also published by the Office for National Statistics [16], and (6) summary data about population health status derived from Clinical Practice Research Datalink (CPRD) Gold [17]. HES APC, HES OPA and ECDS are a set of three event-level, pseudonymised datasets that include detailed information about inpatient, outpatient and emergency department care.

We categorised inpatient spells as elective, non-elective, or maternity using the admission method field. Inpatient maternity activity was limited to females aged 15–49 years. We retained activity associated with well-babies (treatment function 424). We excluded outpatient attendance records where the planned attendance did not occur (codes 2,3,4 and 7). ED attendances were limited to those taking place in type 1 (multi-specialty consultant-led) departments. We excluded all events where the patient was resident outside of England or did not have a valid recorded age and sex. We summarised this dataset as a series of counts of the 5 types of hospital activity, by year, age and sex.

We use mid-year population estimates from the Office for National Statistics to analyse population growth [15]. The mid-year population estimates (MYE) contain information about the numbers of people living in England by single-year-of-age and sex in the middle of each calendar year. Life Tables contain information about period life expectancy by single-year-of age, sex and calendar year. We used MYEs and life tables from each year from 2011 to 2019. All ages over 90 years were grouped together as 90+ years.

To capture information about changes in age-specific health status (H), we used CPRD Gold to estimate the proportion of the population with a Cambridge Multimorbidity Score (CMS) greater than 1.5, stratified by age, sex, and calendar year. The CMS is a validated measure of illness severity that is commonly used to explain variation in and predict levels of healthcare activity and health outcomes [18]. A patient’s CMS score is a function of the presence or absence of 20 forms of morbidity recorded within their primary care record. Weights are assigned to each type of morbidity. In 2019 for those aged 30+ years in England with any illness (CMS greater than 0), nearly a third of all individuals had a score greater than 1.5 (70^th^ percentile) [19]. The mean score was 1.2 and the median score was 0.8. A score of 1.5 has been used by others to indicate ‘major illness’ [20]. We used published estimates of the proportion of the population with a CMS greater than 1.5 by calendar year (2011–2019), single-year-of-age, and sex [21]. The estimates were derived from year-stratified random samples of 20,000 patient records per year, from CPRD Gold, a research dataset comprising a large representative sample of electronic primary care records. Age-specific health status (H) is applied as an averaged effect by age, sex, and year.

Our final analysis dataset took the form of counts of each of the 5 types of hospital activity, the population, remaining years of life expectancy, and the proportion of the population with a CMS score greater than 1.5 for each combination of year (2011–2019), single-year-of-age (0 to 89, and 90+ years), and sex.

### Statistical methods

We begin with descriptive analyses, plotting trends in activity and in the population size, demonstrating changes in the population age-sex-structure and illustrating the relationship between age and activity rates by sex.

### Health-status age

We created a new measure called “health status age” to allow us to reflect changes in an age cohort’s average health status, relative to the actual (chronological) age of the cohort. We calculate this health-status age for each combination of age, sex, and year in our dataset. In the baseline year (2011), and for people younger than 55 years, we assume that health-status age is the same as chronological age. However, for people aged 55 years and over in later years, we allow health-status age to differ—reflecting changes in life expectancy and illness levels since 2011. To do this, we use the Sullivan method [18], which estimates how many years people can expect to live without major illness (also called disability-free life expectancy). We base this on standard life tables, and the proportion of people with a CMS greater than 1.5 (indicating significant illness). The formula we use adjusts a person’s age based on how much disability-free life expectancy has changed since 2011, relative to changes in overall life expectancy. In simple terms, if people are living longer and staying healthier, their health-status age will be younger than their actual age—and vice versa.

Mathematically the health status age associated with chronological age *i* to in year *y* can be expressed as:

$$\begin{aligned}health\; status\; ag{e_{i\; to\; y\; to\; f|m}} = i - (dfl{e_{i\; to\; y\; to\; f|m}} - dfl{e_{i\; to\; y\; = 2011\; to\; f|m}})\\\left( {{{l{e_{i\; to\; y\; to\; f|m}} - l{e_{i\; to\; y\; = 2011\; to\; f|m}}} \over {l{e_{i\; =\; 65\; to\; y\; to\; f|m}} - l{e_{i\; =\; 65\; to\; y\; =\; 2011\; to\; f|m}}}}} \right)\end{aligned}$$

where:

$${\rm{i}} \ge 55\,{\rm{ is\, chronological\, age}}$$ in years

$$2011{\rm{ }} < {\rm{y }} \le 2019\;{\rm{ is\; calendar\; year}}$$

$${\rm{l}}{{\rm{e}}_{{\rm{i\; to\; y\; to\; f}}|{\rm{m}}}}{\rm{ is\; life\; expectancy \;at\; age\; i \;to\; sex\; f}}|{\rm{m\; to\; in\; year\; y}}$$

$${\rm{dfl}}{{\rm{e}}_{{\rm{i\; to\; y\; to\; f}}|{\rm{m}}}}{\rm{ is\; disability\; free\; life\; expectancy\; at\; age\; i\; to\; sex\; f}}|{\rm{m\; to\; in\; year\; y }}$$

and disability-free is taken as having CMS < 1.5

### Decomposing activity growth

We posit that the annual growth rate in the counts of hospital activity is given by the following growth equation below:

$$G = \left( {1 + P} \right)\left( {1 + S} \right)\left( {1 + H} \right)\left( {1 + R} \right) - 1 $$

where:

G is total growth in hospital activity

P is growth factor due to changes population size

S is growth factor due to changes in the age-sex profile of the population

H is growth factor due to changes in the health status of the population

R is the residual growth factor

We use general-additive negative binomial regression models with splines to estimate growth in hospital activity (counts) by year as shown in the table below based on the overall notion that growth in counts of hospital activity is the product of four factors – population size, population age-sex profile, population age specific health status and some residual effect. Each model in the table is applied to each point of delivery. The exponentiated coefficient for year variable (2011 to 2019) is the annual growth rate.

**Table:** 

Model	Model description (pseudo R code)
M1	activity ~ β_M1_year
M2	activity ~ β_M2_year + offset(log(population_size)
M3^†^	activity ~ β_M3_year + s(age, by = sex) + offset(log(population_size)
M4^†^	activity ~ β_M4_year + s(health_status_age, by = sex) + offset(log(population_size)

† - the sex variable is excluded for the maternity model, where β is the coefficient of interest with subscripts showing the model (M1 to M4). {full model results are reported in the supplementary file}

It then follows

- G = exp(β_M1_)
- *p* = exp(β_M1_)/exp(β_M2_)
- S = exp(β_M2_)/exp(β_M3_)
- H = exp(β_M3_)/exp(β_M4_)
- *R* = exp(β_M4_)

The 95% CI for G and R are derived from the distribution of the year coefficient in models M1 and M4.

For P, S, and H, given the central estimates are based on the ratio of two models’ coefficients, we bootstrap the 95% CI by using Monte-Carlo methods. We simulate each of the coefficients β_M1,_ β_M2_, β_M3_, and β_M4_ 1,000 times then view the distribution of the sampled ratios, according to the equations above, and extract the 0.025 and 0.975 quantiles. The central estimates for these growth factors remain those derived algebraically. Given that maternity admissions are restricted to females between the ages of 15 and 49 years and H is defined as applying to those over the age of 55 years, we disregard H in this point of delivery.

The R scripts for our analyses are available via our private GitHub repository (https://github.com/The-Strategy-Unit/non-demographic-growth/tree/main).

## Results

Table 1 shows an overview of the study population. Between 2011 and 2019, the population of both males and females in the dataset steadily increased, with the total population rising from 53.1 million to 56.3 million. The population also aged over time, with the mean age increasing from 39.3 to 40.2 years, and females consistently older than males. The proportion of individuals with a complex morbidity score (CMS > 1.5) showed a slight overall rise, remaining higher in females. Healthcare activity increased across all major categories: elective and non-elective hospital admissions, outpatient attendances, and emergency department visits—all showing growth, especially outpatient visits which rose from 62.9 million to 89.4 million. Females consistently had higher rates of elective admissions and outpatient attendances, while males had slightly more ED attendances. Maternity admissions declined from 1.2 million to 1.1 million.

**Table 1:** Study population characteristics from 2011 to 2019

Characteristic	Sex	Year 2011	Year 2012	Year 2013	Year 2014	Year 2015	Year 2016	Year 2017	Year 2018	Year 2019
Population	Female	26,974,007	27,160,281	27,331,848	27,543,422	27,757,041	27,967,147	28,138,377	28,309,236	28,459,130
	Male	26,133,162	26,333,448	26,533,969	26,773,196	27,029,286	27,300,920	27,481,053	27,667,942	27,827,831
	All	53,107,169	53,493,729	53,865,817	54,316,618	54,786,327	55,268,067	55,619,430	55,977,178	56,286,961
Mean age (years)	Female	40.31	40.39	40.48	40.59	40.66	40.75	40.86	40.97	41.14
	Male	38.30	38.42	38.53	38.66	38.76	38.86	38.99	39.12	39.29
	All	39.32	39.42	39.52	39.64	39.72	39.82	39.94	40.06	40.22
Proportion with CMS > 1.5 (*n* = sample size)	Female	0.096 (10,173)	0.098 (9,972)	0.102 (10,046)	0.102 (10,019)	0.104 (10,152)	0.108 (10,076)	0.095 (9,929)	0.101 (10,035)	0.1 (10,104)
	Male	0.078 (9,791)	0.074 (10,012)	0.086 (9,927)	0.085 (9,944)	0.086 (9,822)	0.089 (9,867)	0.081 (9,991)	0.078 (9,920)	0.083 (9,884)
	All	0.087 (19,964)	0.086 (19,984)	0.094 (19,973)	0.093 (19,963)	0.095 (19,974)	0.098 (19,943)	0.088 (19,920)	0.089 (19,955)	0.092 (19,988)
Elective admission	Female	4,554,054	4,658,159	4,740,056	4,958,696	5,127,589	5,196,314	5,265,462	5,268,572	5,443,616
	Male	4,329,232	4,426,128	4,472,709	4,636,516	4,778,924	4,865,101	4,916,666	4,954,132	5,094,543
	All	8,883,286	9,084,287	9,212,765	9,595,212	9,906,513	10,061,415	10,182,128	10,222,704	10,538,159
Non-elective admission	Female	2,958,628	3,042,272	3,038,532	3,202,376	3,246,549	3,350,531	3,419,847	3,590,728	3,751,972
	Male	2,764,985	2,832,453	2,835,516	2,960,684	3,015,101	3,107,641	3,174,160	3,324,105	3,446,822
	All	5,723,613	5,874,725	5,874,048	6,163,060	6,261,650	6,458,172	6,594,007	6,914,833	7,198,794
Outpatient attendance	Female	36,590,731	39,021,554	41,507,705	44,008,304	47,019,572	49,238,177	50,228,394	51,174,491	51,683,277
	Male	26,323,574	27,978,891	30,021,755	31,895,253	34,033,178	35,720,865	36,507,239	37,352,199	37,678,593
	All	62,914,305	67,000,445	71,529,460	75,903,557	81,052,750	84,959,042	86,735,633	88,526,690	89,361,870
ED attendance	Female	6,402,918	6,873,526	6,907,540	7,164,949	7,212,708	7,662,747	7,602,907	7,620,470	7,774,622
	Male	6,753,429	7,028,372	7,024,635	7,210,164	7,234,212	7,589,819	7,502,762	7,444,823	7,519,318
	All	13,156,347	13,901,898	13,932,175	14,375,113	14,446,920	15,252,566	15,105,669	15,065,293	15,293,940
Maternity admission	Female	1,249,542	1,252,832	1,188,739	1,177,235	1,187,530	1,182,638	1,165,674	1,133,897	1,108,986

**Fig. 1:**
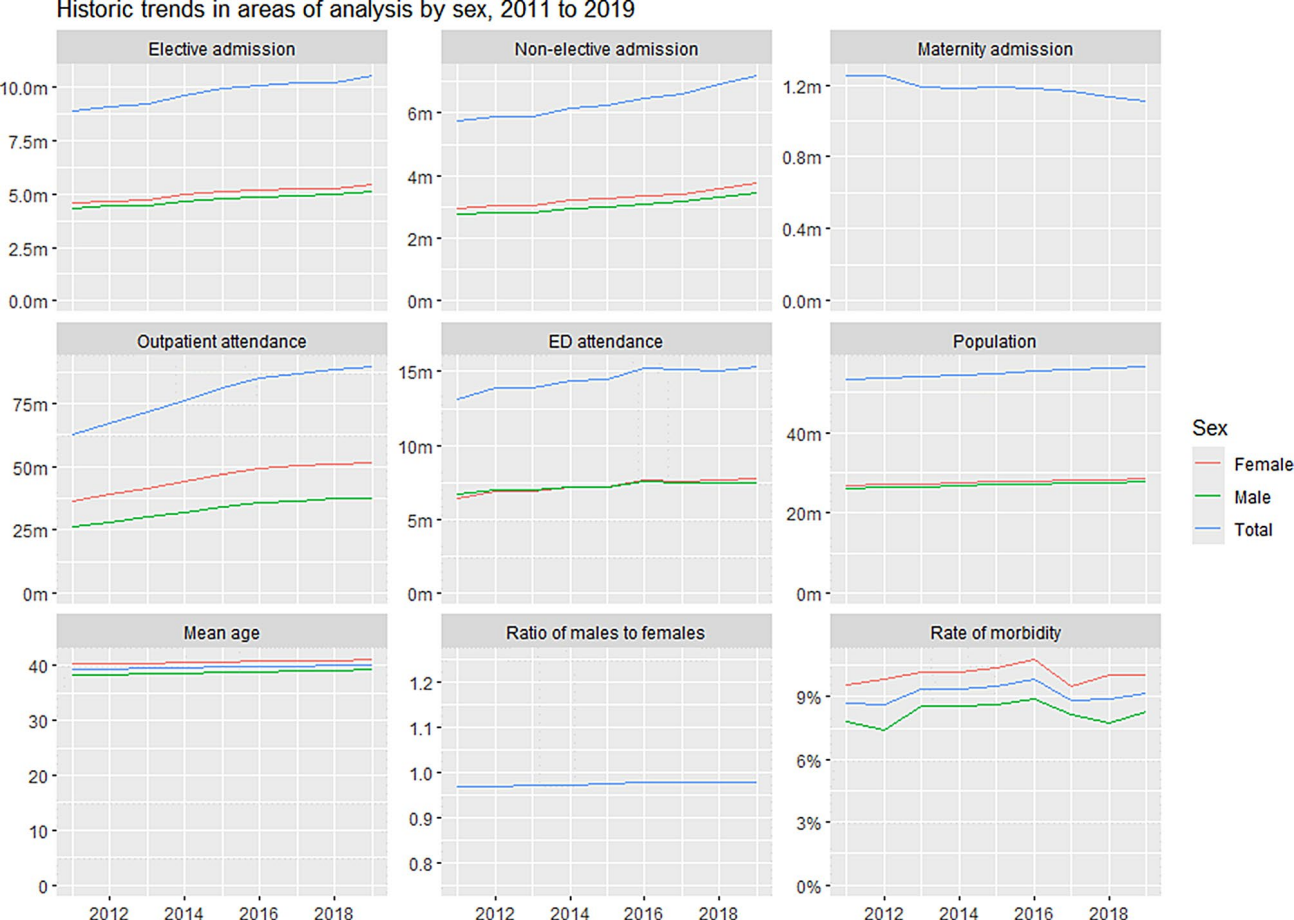
Trends in England from 2011 to 2019. The top row shows (left panel) elective admissions, (middle panel) non-elective admissions and (right panel) maternity admissions. The middle row shows (left panel) outpatient attendances, (middle panel) ed attendances and (right panel) total population. The bottom row shows (left panel) mean ages (years), (middle panel) ratio of male to females and (right panel) percentage of the population with morbidity (CMS score > 1.5)

Figure 1 below shows an overview of the trends, from 2011 to 2019, in England for the variables of interest by sex (where applicable). The top row shows (left panel) the growth in elective admissions, (middle panel) the growth in non-elective admissions and (right panel) the decline in maternity admissions. The middle row shows (left panel) the growth in outpatient attendances, (middle panel) ED attendances and (right panel) total population. The bottom row shows (left panel) trend in mean ages, (middle panel) ratio of male to females and (right panel) percentage of the population with morbidity (CMS score > 1.5). Females were on average, older, had high levels of morbidity and higher levels of elective admissions, non-elective admission and outpatient attendances.

We used general-additive negative binomial regression models to determine each variable in our growth equation. Table 2 below shows the decomposition of total growth (G) by five points of delivery, as per our growth equation:

**Table 2:** The decomposition of total growth (**g**) by five points of delivery, as per our growth equation

Point of delivery	Total growth	Growth attributable to changes in population size	Growth attributable to changes in the population age-sex structure	Growth attributable to changes in the age-specific population health status	Growth attributable to residual, non-demographic, changes
POD	G	P	S	H	R
Elective admission	2.29% (1.06% to 3.54%)	0.88% (−0.96% to 2.67%)	0.69% (−0.62% to 2.18%)	−0.03% (−0.23% to 0.17%)	0.73% (0.58% to 0.88%)
Non-elective admission	2.69% (1.55% to 3.83%)	0.59% (−1.08% to 2.50%)	0.25% (−1.17% to 1.61%)	0.18% (−0.43% to 0.81%)	1.65% (1.13% to 2.17%)
Maternity admission	−0.70% (−5.65% to 4.51%)	0.41% (−6.25% to 8.36%)	−0.08% (−4.93% to 5.23%)	0.00% (NA)	−1.02% (−1.61% to −0.43%)
Outpatient attendance	4.51% (3.62% to 5.40%)	0.61% (−0.71% to 1.96%)	0.46% (−0.63% to 1.56%)	−0.02% (−0.43% to 0.38%)	3.41% (3.11% to 3.71%)
ED attendance	1.29% (0.71% to 1.88%)	0.27% (−0.56% to 1.09%)	0.09% (−0.55% to 0.70%)	0.09% (−0.21% to 0.39%)	0.84% (0.58% to 1.10%)

Total growth rate (G) ranged from −0.70% (−5.65% to 4.51%) (maternity admission) to 4.51% (3.62% to 5.40%) (outpatient attendance). Growth attributable to population size (P) ranged from 0.27% (−0.56% to 1.09%) (ED attendance) to 0.88% (−0.96% to 2.67%) (elective admission). Growth attributable to changes in the population age-sex structure (S) ranged from −0.08% (−4.93% to 5.23%) (−maternity admission) to 0.69% (−0.62% to 2.18%) (elective admission). Growth attributable to changes in the age-specific population health status (H) ranged from −0.03% (−0.23% to 0.17%) (elective admission) to 0.09% (−0.21% to 0.39%) (ED attendance). Growth attributable to residual factors (R) ranged from −1.02% (−1.61% to −0.43%) (maternity admission) to 3.41% (3.11% to 3.71%) (outpatient attendance).Residual (R) growth factors appeared to have the largest impact for all points of delivery to with the exception of elective admissions where P (0.88%) was higher than R (0.73%).

**Fig. 2:**
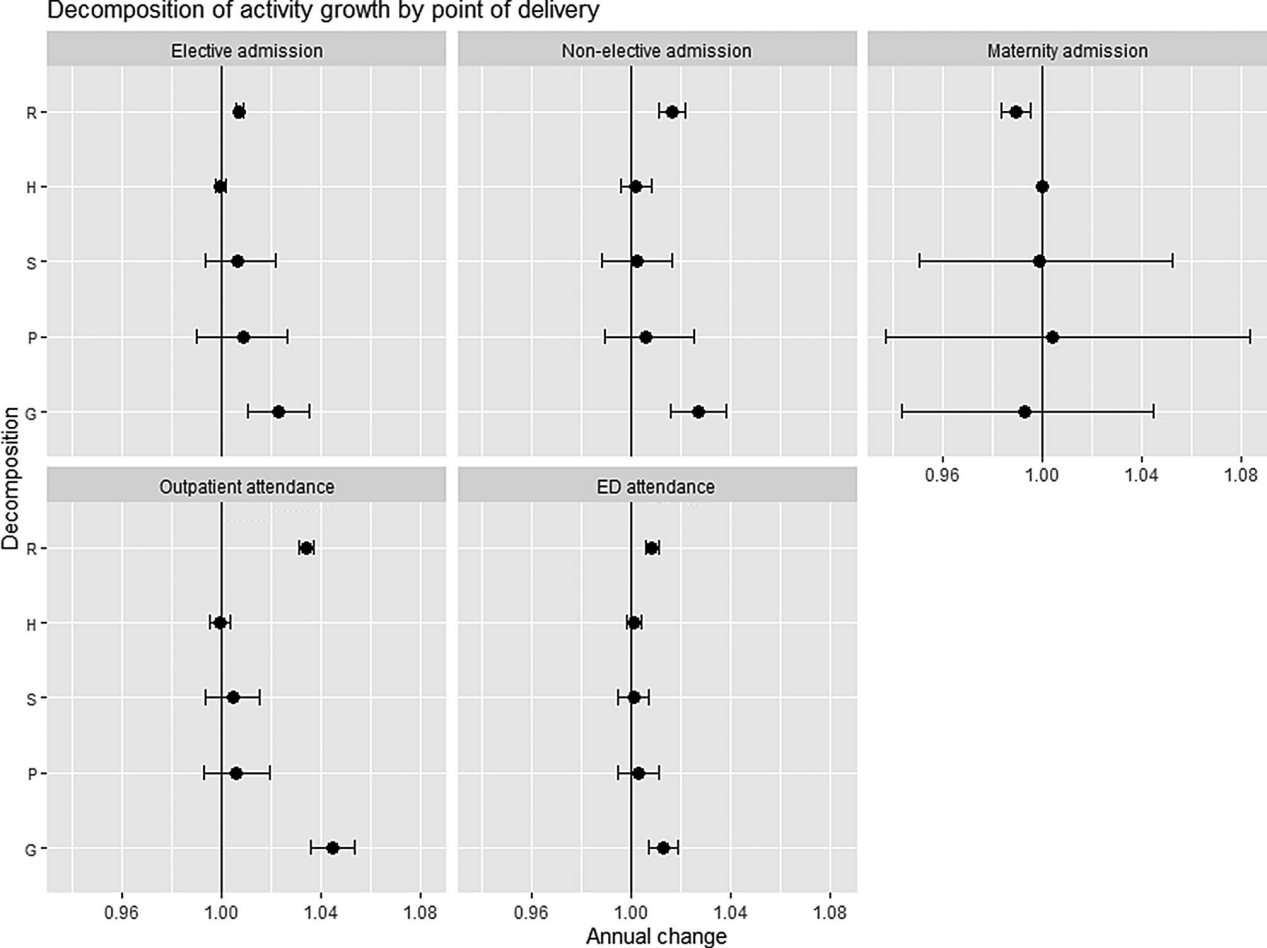
The decomposition of total growth (**g**) by five points of delivery, as per our growth equation based on results from Table 2, where P is growth factor due to changes population size, S is growth factor due to changes in the age-sex profile of the population, H is growth factor due to changes in the health status of the population and R is the residual growth factor

To aid visualisation, the results are plotted in Fig. 2 below. Most components of the growth equation, show in the figure above, cluster around 1. In each panel, the residual (R) appears to be the major driver of total growth.

## Discussion

We sought to provide retrospective estimates of the specific effects of population growth (P), changes in the age-sex profile of the population (S), shifts in population age-specific health status (H) and residual (R) factors on total growth in hospital activity (G), (where G=(1+P)(1+S)(1+H)(1+R)-1) separately for inpatient, maternity, and outpatient activity in English hospitals using data from 2011 to 2019. Overall, we found that only maternity admissions declined while the other types of hospital activity grew. The largest annual growth rate was for outpatient attendance (4.51%) followed by non-elective admission (2.69%) to elective admission (2.20%) and ED attendance (1.29%). In each case the residual (R) growth accounted for the largest or second largest driver of growth. Health status (H) appeared to have relatively minimal impact.

Our findings of growth in hospital activity and reductions in maternity admissions align with those reported by Naser [5], based on earlier data from England and Wales (1999–2019). While Naser’s study accounted for population changes, it did not consider health status.

In our analysis, we found that age-specific health status (H) had the least impact. This finding aligns with longstanding research demonstrating that need alone does not fully explain care use. Andersen’s behavioural model emphasises that utilisation is shaped not only by health needs but also by predisposing and enabling factors, such as socioeconomic status and access to services [8]. Moreover, as Roemer [22] and others continue to show [23], the availability of services often drives hospital activity, independent of demand. In contexts where access is uneven or supply is constrained, health status may be insufficient to predict utilisation. The limited impact of health status in our study reflects the broader patterns in the literature: structural and systemic factors frequently mediate the relationship between health needs and actual service use [24–26].

In our analysis, the residual (R) term functions as a “black box,” capturing the combined influence of factors not explained by population growth (P), demographic shifts (S), or health status changes (H). These include demand-side factors, supply-side factors and broader contextual factors such as policy, service design, or access barriers. The complexity of R reflects the multifaceted nature of healthcare systems and the interplay between supply and demand [27]—particularly the phenomenon of supply-induced demand [28]. Whilst previous studies, across the globe, have often estimated growth in hospital activity [29–32] after accounting for demographic shifts, the role of residual non-demographic factors has received less attention and yet appears to be an important driver of demand [33, 34]. Astolfi et al. [10], in their review of 25 models for forecasting healthcare expenditure (which also included healthcare activity), identified several key drivers of healthcare activity. Such factors include demographic changes, health status, healthcare funding, consumer behaviour, treatment practices (care intensity), technological advancements (medical progress), health productivity (efficiency), and healthcare system organization. In our case it is also worth highlighting that unmet need, typically in the form of patients on long waiting lists and supply side factors such as efficiency gains such as reduction in length of stay, or shifts in care from inpatients to outpatients, are also absorbed in the residual term (R) further complicating its interpretation. Nevertheless, despite the diverse modelling approaches reviewed by Astolfi et al. [10], a key general finding was that the residual (R) plays a significant role in shaping healthcare activity beyond the effects of demographic factors.

Our macro population level longitudinal study has several limitations which should be addressed in future work. Our growth equation has four terms (P, S, H and R). It may be that additional terms are required which currently are subsumed within the residual term (R). For example, the growth equation does not explicitly incorporate factors aimed at mitigating hospital activity via prevention, redirection/substitution or de-adoption of specific types of hospital activity. Such a term would appear as (1-M) in the growth equation. We are investigating the impact of mitigation activities in English hospitals to determine M retrospectively.

In our study, health status was based on the CMS [19], which is a relatively recent tool, that uses common pre-existing conditions to predict death, hospital admission and primary care consultation rate at an individual level. The developers of CMS consider it to be an “optimal approach to describe or adjust for the general health status of individuals in health services and outcomes research” whilst demonstrating that it was superior to the Charlson Comorbidity Index [19]. Nevertheless, the CMS lacks sensitivity to disease severity, patient-reported outcomes, and may not generalise well beyond the development cohort [35]. Moreover, since our health status adjustment is applied as an averaged effect by age, sex, and year it may therefore obscure potentially heterogeneous changes in health status over time, by for example, deprivation, ethnicity, or those with specific health conditions. Future studies should explore alternative measures of health status, notwithstanding the general finding in the literature that changes in age-specific health status has little impact on healthcare utilisation [31, 34].

Although we have analysed the growth equation by five points of delivery, we have not investigated how the growth equation behaves by specific medical conditions which may be useful for planning specific subtypes of hospital activity. For example, the effect of aging varies by medical condition and so services heavily used by elderly patients such as those related to circulatory system diseases (e.g. to heart failure) will see higher growth rates. Future studies could examine how our growth equation behaves in different geographic regions and across populations with different socio-economic profiles, whilst also incorporating supply side factors such as such as beds per capita. Our decomposition model assumes relatively stable relationships among its components based on data from 2011 to 2019, which excludes the shock of covid19 pandemic. Including pandemic years would violate this assumption, as the “residual” (R) would absorb the pandemic shock—making it uninformative or misleading for interpreting underlying trends. Future work could investigate the utility of incorporating the covid19 pandemic into the growth model. Finally, while our findings offer valuable historical empirical evidence, further research is needed to determine how they can inform the planning of future hospitals.

Our study was not designed to unpack the mechanisms by which P, S, H and R causally impact on hospital activity which are known to be multifactorial and complex. This would require a different approach. Astolfi et al. [10] found five studies used a macro-level approach like ours, five used micro-simulation (which requires individual level data), twelve used component-level models (which focus on subgroups to such children to or end of life care) and three used a newer combined approach, which integrate multiple methods (e.g. to combining micro-simulation and macro-level models). These newer combined approaches have the potential to provide a more holistic approach albeit with more intensive data requirements and modelling complexity.

## Conclusions

This ecological study provides retrospective estimates of the specific contributions of population growth (P), changes in the age-sex profile (S), shifts in age-specific health status (H), and residual (R) factors to hospital activity between 2011 and 2019. The total annual growth (G) is modelled as:

G=(1+P)(1+S)(1+H)(1+R)−1. In general, growth in hospital activity was mostly driven by residual (R) factors, followed by population growth (P). Age-specific health status (H) had the lowest impact. These data may provide useful context for planning future hospital activity in England.

## Electronic supplementary material

Below is the link to the electronic supplementary material.

Supplementary Material 1

## Data Availability

The data and analyses that support the findings of this study are available on request from the corresponding author subject to approval by the data owner (NHS England).

## References

[1] Tallack C, Charlesworth A, Kelly E, McConkey R, Rocks S. The bigger picture: learning from two decades of changing nhs care in England. Health Foundation. 2020

[2] Anne M, Santana R, De Las Nieves I, Aragon A, Jose Monserratt M, et al. Drivers of health care expenditure. Discussion paper. 2019. https://eprints.whiterose.ac.uk/153487/1/CHERP169_drivers_health_care_expenditure_final_report.pdf. CHE Research Paper. Centre for Health Economics, University of York, York, UK

[3] Laudicella M, Li Donni P, Olsen KR, Gyrd-Hansen D. Age, morbidity, or something else? A residual approach using microdata to measure the impact of technological progress on health care expenditure. Health Econ. 2022, Jun;31(6):1184–201 35362244 10.1002/hec.4500 PMC9314678

[4] De la Maisonneuve C, Martins JO. The future of health and long-term care spending. OECD J: Econ Stud. 2015, Mar, 27;2014(1):61–96

[5] Rodriguez Santana I, Aragón MJ, Rice N, Mason AR. Trends in and drivers of healthcare expenditure in the English nhs: a retrospective analysis. Health Econ Rev. 2020, Dec;10:1–1 32607791 10.1186/s13561-020-00278-9 PMC7325682

[6] Sebire NJ, Adams A, Celi L, Charlesworth A, Gorgens M, Gorsky M, Landeg O, Nagasawa Y, Nimako KT, Onoka C, Roder-DeWan S. The future hospital in global health systems: The future hospital within the healthcare system. Int J Health Plann Manage. 2025, May;40(3):741–51 39815953 10.1002/hpm.3891 PMC12045726

[7] Ravaghi H, Alidoost S, Mannion R, Bélorgeot VD. Models and methods for determining the optimal number of beds in hospitals and regions: a systematic scoping review. BMC Health Serv Res. 2020, Mar, 6;20(1):186 32143700 10.1186/s12913-020-5023-z PMC7060560

[8] Andersen RM. Revisiting the behavioral model and access to medical care: does it matter? J Health Soc Behav. 1995, Mar;1:1–0 7738325

[9] Babitsch B, Gohl D, Von Lengerke T. Re-revisiting Andersen’s behavioral model of health services use: a systematic review of studies from 1998–2011. GMS Psycho-Soc-Med. 2012, Oct, 25;9:Doc11 10.3205/psm000089 PMC3488807 23133505

[10] NHS England. Nhs in numbers today. https://www.england.nhs.uk/nhsbirthday/about-the-nhs-birthday/nhs-in-numbers-today/. accessed Nov 2023

[11] National Audit Office. Progress with the New hospital programme. In: Department of health & social Care, nhs England. London: Stationery Office; 2023

[12] Department of Health and Social Care Media Centre. RAAC in the nhs – media fact sheet. 2023. https://healthmedia.blog.gov.uk/2023/09/01/media-fact-sheet-raac-in-the-nhs/

[13] NHS England Digital. Hospital episode statistics (hes). https://digital.nhs.uk/data-and-information/data-tools-and-services/data-services/hospital-episode-statistics. Accessed October 29, 2024

[14] NHS England Digital. Emergency Care Data Set (ECDS). https://digital.nhs.uk/data-and-information/data-collections-and-data-sets/data-sets/emergency-care-data-set-ecds. Accessed 24 July 2025

[15] Office for National Statistics. Estimates of the population for the Uk. https://www.ons.gov.uk/peoplepopulationandcommunity/populationandmigde-adopt/populationestimates/datasets/populationestimatesforukenglandandwalesscotlandandnorthernireland. accessed November 2023, England, Wales, Scotland and Northern Ireland

[16] Office for National Statistics: Single-Year Life Tables, Uk: 1980 to 2020 - office for national statistics. Accessed October 31, 2024

[17] Primary Care Data for Public Health Research. https://www.cprd.com/primary-care-data-public-health-research. September 2, 2024

[18] Payne R, Mendonca S, Elliott M, Saunders C, Edwards D, Marshall M, Roland M. Development and validation of the cambridge multimorbidity score. Can Med Assoc J. 2020;192(5):E107. 10.1503/cmaj.190757 32015079 10.1503/cmaj.190757 PMC7004217

[19] Watt T, Raymond A, Rachet-Jacquet H A KK. A microsimulation model for multimorbidity in England. 2022, July. https://www.health.org.uk/sites/default/files/2023-07/Working%20paper%20-%20A%20microsimulation%20model%20for%20multimorbidity%20in%20England.pdf. REAL Centre: Working paper

[20] The Health Foundation (REAL Centre). Health in 2040: projected patterns of illness in England. 2023, July. 10.37829/HF-2023-RC03

[21] Wyatt SM. The gap between need and supply of gp practice consultations. Feb 2024. Methods and data sources. https://www.strategyunitwm.nhs.uk/sites/default/files/2025-07/Methods%20and%20data%20sources%20-%20MDSN%20-%20240220v2.pdf

[22] Roemer MI. Bed supply and hospital utilization: a natural experiment. Hospitals. 1961, Nov, 1;35:36–42 14493273

[23] Gaffney A, McCormick D, Himmelstein G, Woolhandler S, Himmelstein DU. Demand and supply drivers of medicare and non-medicare health spending: an analysis of us States, 1991–2019. Int J Multiling Soc Determinants Of Health And Health Serv. 2025, Jan;55(1):55–63 10.1177/27551938241258399 39053017

[24] Cullis JG, Forster DP, Frost CE. The demand for inpatient treatment: some recent evidence. Appl Econ Lett. 1980, Mar, 1;12(1):43–60

[25] van Doorslaer Ek, Van Vliet RC. “A built bed is a filled bed?” an empirical re-examination. Soc Sciamp; Med. 1989, Jan, 1;28(2):155–64 10.1016/0277-9536(89)90143-3 2928825

[26] Müller D, Akmatov MK, von Stillfried DG. Lower ambulatory care availability and greater hospital capacity are associated with higher hospital case volumes. Res Health Serv Regions. 2025, Jun, 10;4(1):7 10.1007/s43999-025-00066-0 PMC12151972 40493273

[27] Jagger C, Cox B, Le Roy S, Clavel A, Robine JM, Romieu I, Van Oyen H. Health expectancy calculation by the Sullivan method: a practical guide. Nihon University population research institute (nupri) research paper series. 2014, Oct, 68

[28] Kumar A, Lam SS, Chan SL, Xu Y, Ge Y, Gui GK, Tan HK. Strategizing towards the future hospital: a systems thinking approach. Health Res Policy Syst. 2025, May, 27;23(1):71 40426118 10.1186/s12961-025-01333-9 PMC12107927

[29] Naser AY. Hospitalisation profile in England and Wales, 1999 to 2019: an ecological study. BMJ Open. 2023, Apr, 1;13(4):e068393 37024246 10.1136/bmjopen-2022-068393 PMC10083742

[30] Pandey A, Ploubidis GB, Clarke L, Dandona L. Hospitalisation trends in India from serial cross-sectional nationwide surveys: 1995 to 2014. BMJ Open. 2017, Dec, 1;7(12):e014188 29259052 10.1136/bmjopen-2016-014188 PMC5770834

[31] Astolfi R, Lorenzoni L, Oderkirk J. Informing policy makers about future health spending: a comparative analysis of forecasting methods in OECD countries. Health Policy (new Y). 2012, Sep, 1;107(1):1–0 10.1016/j.healthpol.2012.05.001 22682763

[32] Australian Institute of Health and Welfare. Top reasons for hospitalisation. 2017. Available: https://www.indigenoushpf.gov.au/measures/1-02-top-reasons-hospitalisation. Accessed 21 Sep 2021

[33] Ministry of Health Singapore. Top 10 conditions of hospitalisation. 2012. Available: https://www.moh.gov.sg/resources-statistics/singapore-health-facts/top-10-conditions-of-hospitalisation

[34] White C. Health care spending growth: how different is the United States from the rest of the OECD?. Health Aff (Millwood). 2007, Jan;26(1):154–61 17211024 10.1377/hlthaff.26.1.154

[35] Harrison H, Ip S, Renzi C, Li Y, Barclay M, Usher-Smith J, Lyratzopoulos G, Wood A, Antoniou AC. Implementation and external validation of the Cambridge Multimorbidity Score in the UK Biobank cohort. BMC Med Res Methodol. 2024, Mar, 20;24(1):71 38509467 10.1186/s12874-024-02175-9 PMC10953059

