# Supplementary material for: Decomposing the effects of changes of population size, age-sex profile, health status and residual factors on growth in hospital activity in English hospitals: an ecological database study from 2011–2019
Source: BMC Health Serv Res. 2025 Dec 17;25:1601. doi: 10.1186/s12913-025-13662-0 (PMC12709711; doi:10.1186/s12913-025-13662-0)
Supplement: Supplementary file 1 — Supplementary Material 1 [file 12913_2025_13662_MOESM1_ESM.docx]

**Supplementary file**

**The table below shows the model coefficients for each of four models for each point of delivery.**

| Point of delivery | model | term | estimate | std.error | statistic | p.value |
| --- | --- | --- | --- | --- | --- | --- |
| Elective admission | model_1 | (Intercept) | -34.8006 | 12.46238 | -2.79245 | 0.005231 |
|  | model_1 | year | 0.022655 | 0.006185 | 3.663033 | 0.000249 |
|  | model_2 | (Intercept) | -29.6926 | 14.61138 | -2.03216 | 0.042138 |
|  | model_2 | year | 0.013878 | 0.007251 | 1.913975 | 0.055623 |
|  | model_3 | (Intercept) | -16.0008 | 1.397577 | -11.449 | 2.38E-30 |
|  | model_3 | year | 0.007009 | 0.000694 | 10.1055 | 5.22E-24 |
|  | model_4 | (Intercept) | -16.5104 | 1.506783 | -10.9574 | 6.13E-28 |
|  | model_4 | year | 0.007262 | 0.000748 | 9.712145 | 2.68E-22 |
| Non-elective admission | model_1 | (Intercept) | -43.0051 | 11.4238 | -3.76452 | 0.000167 |
|  | model_1 | year | 0.026503 | 0.005669 | 4.674856 | 2.94E-06 |
|  | model_2 | (Intercept) | -43.7411 | 14.2201 | -3.07601 | 0.002098 |
|  | model_2 | year | 0.020637 | 0.007057 | 2.924306 | 0.003452 |
|  | model_3 | (Intercept) | -38.8531 | 3.542046 | -10.9691 | 5.38E-28 |
|  | model_3 | year | 0.018165 | 0.001758 | 10.33386 | 4.95E-25 |
|  | model_4 | (Intercept) | -35.1441 | 5.232384 | -6.71666 | 1.86E-11 |
|  | model_4 | year | 0.016333 | 0.002597 | 6.290036 | 3.17E-10 |
| Maternity admission | model_1 | (Intercept) | 24.54175 | 52.5803 | 0.466748 | 0.64068 |
|  | model_1 | year | -0.007 | 0.026095 | -0.26822 | 0.788532 |
|  | model_2 | (Intercept) | 20.05805 | 52.36372 | 0.383052 | 0.701681 |
|  | model_2 | year | -0.01113 | 0.025987 | -0.42821 | 0.668497 |
|  | model_3 | (Intercept) | 17.53679 | 6.116632 | 2.867066 | 0.004143 |
|  | model_3 | year | -0.01028 | 0.003036 | -3.38717 | 0.000706 |
|  | model_4 | (Intercept) | 17.53679 | 6.116632 | 2.867066 | 0.004143 |
|  | model_4 | year | -0.01028 | 0.003036 | -3.38717 | 0.000706 |
| Outpatient attendance | model_1 | (Intercept) | -75.8412 | 8.753507 | -8.66409 | 4.55E-18 |
|  | model_1 | year | 0.044075 | 0.004344 | 10.14605 | 3.45E-24 |
|  | model_2 | (Intercept) | -76.2055 | 10.6094 | -7.18282 | 6.83E-13 |
|  | model_2 | year | 0.037996 | 0.005265 | 7.216554 | 5.33E-13 |
|  | model_3 | (Intercept) | -66.9169 | 2.96326 | -22.5822 | 6.48E-113 |
|  | model_3 | year | 0.033382 | 0.001471 | 22.7 | 4.48E-114 |
|  | model_4 | (Intercept) | -67.2569 | 2.973599 | -22.618 | 2.88E-113 |
|  | model_4 | year | 0.033551 | 0.001476 | 22.73555 | 1.99E-114 |
| ED attendance | model_1 | (Intercept) | -14.5251 | 5.996424 | -2.42229 | 0.015423 |
|  | model_1 | year | 0.012832 | 0.002976 | 4.312005 | 1.62E-05 |
|  | model_2 | (Intercept) | -21.7983 | 6.376465 | -3.41856 | 0.00063 |
|  | model_2 | year | 0.010158 | 0.003164 | 3.210189 | 0.001326 |
|  | model_3 | (Intercept) | -20.0229 | 1.675676 | -11.9492 | 6.56E-33 |
|  | model_3 | year | 0.009287 | 0.000832 | 11.16749 | 5.88E-29 |
|  | model_4 | (Intercept) | -18.1717 | 2.611086 | -6.95943 | 3.42E-12 |
|  | model_4 | year | 0.00837 | 0.001296 | 6.459605 | 1.05E-10 |
